# Association of vitamin D and diarrhoea in children aged less than five years at Muhimbili national hospital, Dar es Salaam: an unmatched case control study

**DOI:** 10.1186/s12887-019-1614-4

**Published:** 2019-07-15

**Authors:** Imran Hassam, Rodrick Kisenge, Said Aboud, Karim Manji

**Affiliations:** 1 Shree Hindu Mandal Hospital, P.O.Box 581, Sewa Street, Dar es Salaam, Tanzania; 2 Department of Paediatrics and Child Health, Muhimbili University College of Health and Allied Sciences, P. O. Box 65001, United Nations Road, Dar es Salaam, Tanzania; 3 Department of Microbiology and Immunology, Muhimbili University College of Health and Allied Sciences, P. O. Box 65001, United Nations Road, Dar es Salaam, Tanzania

**Keywords:** Vitamin D, Diarrhoea, Children, Tanzania

## Abstract

**Background:**

There has been a growing interest in the non-skeletal roles of vitamin D particularly its immune-modulatory properties which has been shown to influence the susceptibility and severity to infections. There is insufficient data globally on the association between Vitamin D levels and Diarrhoea in children. The objective of the study was to determine the association between vitamin D levels and diarrhoea in children aged less than five years.

**Methods:**

Hospital based unmatched case-control study was carried out at MNH between September 2015 and January 2016. Cases were defined as patients with diarrhoea, Sick controls were patients who did not have diarrhoea but were admitted for other illnesses and Healthy controls were children who had neither diarrhoea nor other co-morbid conditions.

Structured questionnaires were used to capture the demographic data and anthropometric measurements. Blood samples of study participants were tested for serum vitamin D levels and grouped as vitamin D sufficient, insufficient or deficient (VDD). SPSSv.20 was used to carry out the Statistical analysis. Binary logistic regression, Mann-Whitney and Kruskal-Wallis tests were used, a *p*-value≤ 0.05 was considered to be statistically significant.

**Results:**

A total of 188 children under five were recruited in the study at the ratio of 1 case: 3 controls, of these 47 were Cases, 94 were Sick controls and remaining 47 were Healthy controls. The mean age was 17.01 ± 14.8 months. The mean vitamin D level was 51.18 ± 21.97 nmol/l. Majority of the participants 101 (53.7%) were vitamin D deficient, 64 (34%) were insufficient and 23 (12.2%) had sufficient vitamin D levels. Sick controls were 3.2 times more likely to be VDD compared to cases [95% CI 0.14–0.69; *p* = 0.0015] and 5.03 times when compared to Healthy controls [95% CI 2.22–11.55; *p* = 0.000]. Severe acute malnutrition (SAM) was independently associated with diarrhoea (95% CI: 1.26–5.39, p 0.01).

**Conclusions:**

High prevalence of vitamin D deficiency was found in the children under five years studied. Vitamin D levels was not found to be specifically associated with diarrhoea in children under five years of age.

## Background

Vitamin D is a fat-soluble vitamin and plays many pivotal roles in body processes and functions. The primary function of vitamin D is to increase the efficiency of absorption of calcium and phosphorus from the small intestine and therefore playing a major role in calcium homeostasis and thus skeletal health. However vitamin D has several other extra skeletal and calcium homeostasis functions, particularly as an immune modulator [1].

Diarrhoea is one of the most common diseases affecting children under the age of five years in developing countries and it is the second most common cause of morbidity and mortality due to an infectious cause in this age group [2, 3].

Low levels of vitamin D are associated with susceptibility and severity of acute infections and with unfavourable outcomes to some chronic infections [4].

There are numerous studies showing the relationship between low vitamin D levels and pneumonia, pulmonary tuberculosis, asthma, otitis media and thoracic empyema [5–8]. However, studies on vitamin D levels and its association with diarrhoea are scarce. A study conducted in Bogota, Columbia showed that vitamin D deficiency was associated with increased incidence of diarrhoea in school aged children [9], likewise three studies carried out in Qalubia governorate, Egypt; Hazara division, Pakistan and in Jeddah, Saudi Arabia showed vitamin D deficiency to be associated with an increased rate of diarrhoea [10–12].

Studies in children have shown there is a high prevalence of vitamin D deficiency in Tanzania. Prospective cohort study in HIV infected and HIV exposed children in Dar es Salaam, Tanzania revealed low level of vitamin D in both groups of an average of 18 ng/mL indicating probable vitamin D deficiency in Tanzanian children [13]. Likewise a separate analysis of the HIV exposed uninfected children from the same study was carried out at 6 months of age and it showed prevalence of vitamin D deficiency of 34.6% [14]. These findings may indicate that children in Tanzania may have vitamin D deficiency which is unrecognized, although these studies cannot be generalised. No study has been carried out to investigate the vitamin D status in general population of children under the age of five years in Tanzania and there isn’t sufficient data globally and locally pertaining to vitamin D status and diarrhoea in under-fives.

This study investigated the association between vitamin D levels among children with diarrhoea compared to children without diarrhoea in a similar setting at Muhimbili National Hospital (MNH).

## Methods

This was a hospital based unmatched case-control study carried out in MNH which is located in Dar es Salaam, Tanzania. The study was carried out from September 2015 to January 2016.

The case control study was designed at a ratio of 1:3 for Cases: Control. Cases included all children under five years of age who had only diarrhoea as the main morbidity. The controls were grouped into two, sick controls and healthy controls. Sick controls were children who were admitted due to other morbidities and not having diarrhoea, and healthy controls, were not having any morbidity and were recruited from the routine childhood clinics.

The ratio of children in the sub grouping was 1:2:1 i.e. Cases: Sick Controls: Healthy Controls.

The inclusion criteria for the cases were all children under the age of five with diarrhoea who had been admitted in the diarrhoea ward at MNH. For the controls; children of the same group admitted due to other co-morbid conditions apart from diarrhoeal disease, those who had not suffered an episode of diarrhoea in the past four weeks prior to admission or were children attending follow-up OPD clinic or vaccination clinic who had neither diarrhoea nor other co-morbid conditions. As determined by looking at patient’s clinical notes and/or questioning the parent/guardian.

Children excluded from the study were those; with suspected renal disorder or liver disease which was determined by history, clinical features, relevant laboratory results and by patient’s case notes; who had received vitamin D or multi vitamin supplements; who were on Phenytoin, Phenobarbitone or Rifampicin as treatment or had undergone small intestine resection; had cerebral palsy or whose parents/guardians did not provide an informed written consent.

The minimum sample size was determined using the Kelsey formula for unmatched case control studies. The proportion for cases was derived from a study carried out in Egypt that showed that in patients with recurrent acute diarrhoea, vitamin D deficiency was found to be 58% [10]. Although the study involved patients with recurrent acute diarrhoea, it was among the few conducted in Africa and hence it was used to calculate the sample size. The proportion for the controls, the closest data that is available is that among HIV exposed uninfected children in Tanzania that showed a prevalence of 34.6% [14]. Studies have also shown that HIV exposed uninfected children have a higher prevalence of VDD with an average of 18.1 ng/ml [13]. Based on this, a figure of 30% was selected as the proportion for the controls.

Consecutive sampling was employed and all under five patients admitted or were attending the out-patient follow up or vaccination clinic and who meet the inclusion criteria.

### Data collection

The basic and demographic information of each was recorded. All information was recorded in a Case Record Form designed for the study. Anthropometric measurement of children included; Weight, Height/length and Mid Upper Arm Circumference (MUAC). All children were weighed using a standard SECA™ beam balance and weight was recorded to the nearest 10 g. MUAC was measured using a tape measure at the mid-point of the less dominant arm and recorded in the case record form. Length was recorded to the nearest 1 cm using either length boards of height stands depending on the age of the child. WHO anthropometric charts were used to classify children as having Normal nutrition status, Mild, Moderate or Severe acute malnutrition using Z scores. Research assistants were all doctors who had been trained on administering the questionnaire and on the technique of collecting blood samples and anthropometric measurements.

Blood samples for vitamin D testing were taken within 48 h of admission. The blood samples of those seen at OPD were taken immediately after completion of the questionnaire.

Two millilitres of whole blood was collected from the anterior cubital fossa after swabbing the area with 70% methylated alcohol/spirit and allowing it to fully dry before carrying out the blood draw. Blood was immediately put in a red capped vacutainer. Gentle mixing of the specimen bottle to avoid haemolysis was ensured. The blood samples were then transported to the laboratory within 2 h of collection. In the lab, the samples were centrifuged to obtain the serum. The serum sample was then aliquoted and stored at a temperature of − 86° Celsius until a set number of samples had been accumulated to run a batch test. Vitamin D levels were measured using 25(OH) EIA™ (Immunodiagnostics, Tyne & Wear, UK. Product number AC-57SF1). In every run, seven calibrators (calibration 0–6) and two levels of controls were included and run in duplicates. Assay run was considered valid if all 7 calibrators and controls were within the acceptable range.

Laboratory technicians were blinded to the diarrhoea status of the patients’ in-order to avoid bias while testing. Testing were carried out at the Muhimbili University College of Health and Allied Sciences (MUHAS) Clinical Research Lab which participates in vitamin D External Quality Assessment Scheme (DEQAS), which is a quality assurance program, to ensure accuracy and reliability of the results.

### Data management and analysis

The data collected were recorded in the case report forms and entered in to a computer data base SPSS windows Version 20.0.

The SPSS was used to carry out statistical analysis. Representation of data was done by using means and standard deviations for normally distributed numerical data and medians and interquartile range for non-normally distributed numerical data. Proportions were used for categorical data.

The Mann-Whitney test was used to compare medians of vitamin D levels between different groups. The Kruskal-Wallis test was used to test the difference in median serum vitamin D levels between all three groups (i.e. cases, sick controls and healthy controls). Chi square test was used to determine associations of different categorical variables between the cases and controls. Odds ratios were used as a measure of association. Binary logistic regression analysis was done to determine independent association between the dependent (diarrhoea in children) and independent variables (socio-demographic and clinical variables such as nutritional status). All variables with *p* value < 0.2 were included in the regression model. A *p*-value of less than to 0.05 was considered statistically significant.

The primary outcome was association between vitamin D status and diarrhoea. Secondary outcomes were associations between diarrhoea and other independent variables. Secondary outcomes were assessed to see if these may have confounded the results for the primary outcome.

A separate group analysis was conducted to compare healthy controls and sick controls. This was done to determine if presence of co-morbidity affected the vitamin D status in the controls.

## Results

A total of 188 children under the age of five were included in the study of which 47 were cases, 94 were Sick controls and 47 were Healthy controls. The mean age of the participants was 17.01 ± 14.80 months. Of these, 47 (25%) were Cases (had diarrhoea); 47 (25%) were Healthy controls without any other co-morbidities and 94 (50%) were Sick controls i.e. controls with other co-morbid condition. The mean age of cases, sick controls and healthy controls were 15.51 ± 9.07 months, 19.80 ± 16.70 months and 12.94 ± 14.48 months, respectively. The mean vitamin D level was 51.18 ± 21.97 nmol/L.

Common illness among the cases (apart from diarrhoea) and sick controls were malaria, sickle-cell disease, malnutrition and other infectious diseases.

There was a higher predominance of males (70.2% among cases, 53.2% controls) and majority of the study population were 12 months of age; had a birth weight equal to or above 2.5 kg or had been exclusively breast fed. Among the cases, there were one third who had normal nutritional status, one third were severely malnourished and one third had mild-moderate malnutrition (Table 1). Maternal age, religion, educational status and marital status and occupation were of similar magnitude among cases and controls. Majority of the fathers were; 25 years of age or younger and had similar primary level of education and were working as skilled labourers.

**Table 1:** Baseline Demographic Characteristics of the Study Participants

Child Characteristic	Case (*n* = 47)	Control (*n* = 141)	Total (*n* = 188)	P Value
1. Sex.				
Male	33 (70.2%)	75 (53.2%)	108	
Female	14 (29.8%)	66 (46.8%)	80	0.06
2. Age (Months).				
≤ 12	23 (48.9%)	68 (48.2%)	91	
> 12	24 (51.1%)	73 (51.8%)	97	1.00
3. Birth Weight (Kg).				
< 2.5	8 (17.0%)	15 (10.6%)	23	
≥ 2.5	39 (83.0%)	119 (89.4%)	158	0.315
4. Exclusive Breast Fed.				
Yes	21 (44.7%)	71 (50.4%)	92	
No	26 (55.3%)	70 (49.6%)	96	0.614
5. Nutritional Status.				
Normal	15 (31.9%)	74 (52.5%)	89	
Mild Acute Malnutrition	8 (17.0%)	24 (17.0%)	32	
Moderate Malnutrition	9 (19.2%)	20 (14.2%)	29	
Severe Acute Malnutrition	15 (31.9%)	23 (16.3%)	38	0.046

Apart from the number of diarrhoea episodes in the past year, nutritional status of the child and paternal education, there were no differences between the demographic characteristics among the cases and controls highlighting the fact that the selection is not biased (Table 1).

Of the 188 participants, majority 101 (53.7%) were found to be vitamin D deficient, 64 (34.0%) were found to be insufficient and 23 (12.2%) had sufficient levels of vitamin D. There was a higher male predominance for vitamin D deficiency 61 (60.3%) than female 40 (39.7%) [OR 1.3, [95% CI 0.43–1.38; *p* = 0.77].

The Mann-Whitney test showed no significant difference in the median vitamin D levels between the cases and controls. The median vitamin D level for the cases was 51.93 nmol/L, IQR 16.98 nmol/L and for the controls was 47.79 nmol/L, IQR 20.47 nmol/L (*p* value = 0.092).

The Kruskal-Wallis test was used to test the difference in median serum vitamin D levels between all three groups (i.e. cases, sick controls and healthy controls) and revealed a statistically significant difference between the three groups in their median serum vitamin D levels (*p* < 0.001). Post hoc analysis revealed that there were statistically significantly lower median serum vitamin D levels in the sick controls (44.7 nmol/L, IQR 19.92 nmol/L) when compared to the cases (*p* = 0.008) and also when compared to the healthy controls (55.74 nmol/L, IQR 27.52 nmol/L) (*p* < 0.001). There was no statistically significant difference between the cases and healthy controls (*p* = 0.263).

Vitamin D levels were re-categorized for analysis purpose as *Sufficient* (to include both sufficient and insufficient vitamin D levels **-**the grouping was necessary due to the low numbers of patients with sufficient vitamin D levels) and *Deficient* (those with vitamin D deficiency).

Out of a total of 188 participants, majority (57.4%) of the children in the control group were vitamin D deficient while majority (57.4%) of the cases were vitamin D sufficient. The odds of VDD in All controls was 1.8 times compared to Cases, however this finding was not statistically significant (Table 2).

**Table 2:** Vitamin D Status between Cases and Controls

	Vitamin D Status		Total	
	Deficient	Sufficient		
Diarrhoea*	20 (42.6%)	27 (57.4%)	47	OR 0.55 (95% CI 0.27–1.13) *P* = 0.092
No diarrhoea	81 (57.4%)	60 (42.6%)	141	
Total	101 (53.7%)	87 (46.3%)	188	

*Diarrhoea being cases; No diarrhoea being controls

Sub-analysis between Cases and Sick controls, showed that Cases had 69% less likely chance of having vitamin D deficiency compared to the Sick controls. (OR 0.31 [95% CI 0.14–0.69]; *p* = 0.0015).

Similar sub-analysis between Cases and Healthy controls, showed that Cases were 1.6 times more likely to have vitamin D deficiency when compared to Healthy controls, however this finding was not statistically significant.

On the other hand, comparison within controls (sick controls versus healthy controls) showed that odds of vitamin D deficiency in sick controls was 5.03 times compared to healthy controls [95% CI 2.22–11.55; *p* = 0.000]. (Table 3). However, the study was not powered enough to look at comparison between the two groups of control.

**Table 3:** Vitamin D Status between Sick Controls and Healthy Controls. (Sub-group Analysis within Control Group)

	Vitamin D Status		Total	
	Deficient	Sufficient		
Sick Controls	66 (70.2%)	28 (29.8%)	94	OR 5.03 (95% CI 2.22–11.55) P = 0.000
Healthy Controls	15 (31.9%)	32 (68.1%)	47	
Total	81 (57.4%)	60 (42.6%)	141	

Cases and controls were compared based on their vitamin D status to different factors. Tables 4 and 5 show the relationship between different socio-demographic factors among the cases and controls in vitamin D deficient and vitamin D sufficient children, respectively.

**Table 4:** Socio-demographic characteristics in relation to Vitamin D deficiency among cases and controls

Characteristic	Vitamin D Deficient		Total	OR (95% CI), p value
	Cases	Controls		
1.Socioeconomic Status.				
1st Tertile	4 (20.0%)	23 (28.4%)	27 (26.7%)	*P* = 0.697
2nd Tertile	11 (55.0%)	37 (45.7%)	48 (47.5%)	
3rd Tertile	5 (25.0%)	21 (25.9%)	26 (25.7%)	
Total	20	81	101	
2. Maternal Education Level.				0.93 (0.34–2.53) *p* = 0.887
Informal/Primary	12 (60.0%)	50 (61.7%)	62 (61.4%)	
Secondary/Higher	8 (40.0%)	31 (38.3%)	39 (38.6%)	
Total	20	81	101	
3. Maternal Occupation.				0.72 (0.27–1.94) *p* = 0.513
Not Working	8 (40.0%)	39 (48.1%)	47 (46.5%)	
Working	12 (60.0%)	42 (51.9%)	54 (53.5%)	
Total	20	81	101	
4.Maternal Age (Years)				1.25 (0.47–3.35) *P* = 0.653
≤ 28	11 (55.0%)	40 (49.4%)	51 (50.5%)	
≥ 29	9 (45.0%)	41 (50.6%)	50 (49.5%)	
Total	20	81	101	
5. Maternal Religion.				1.78 (0.62–5.09) *P* = 0.281
Muslim	14 (70.0%)	46 (56.8%)	60 (59.4%)	
Christian	6 (30.0%)	35 (43.2%)	41 (40.6%)	
Total	20	81	101	
6.Maternal Marital Status.				*P* = 0.118
Married/Cohabiting	20 (100.0%)	72 (88.9%)	92 (91.1%)	
Single/Divorced	0	9 (11.1%)	9 (8.9%)	
Total	20	81	101

**Table 5:** Socio-demographic characteristics in relation to Vitamin D sufficiency among cases and controls

Characteristic	Vitamin D Sufficiency		Total	OR (95% CI), p value
	Cases	Controls		
1. Socioeconomic Status.				
1st Tertile	5 (19.2%)	21 (80.8%)	26	*P* = 0.160
2nd Tertile	11 (30.6%)	25 (69.4%)	36	
3rd Tertile	11 (44.0%)	14 (56.0%)	25	
Total	27 (31.0%)	60 (69.0%)	87	
2. Maternal Education Level.				0.50 (0.18–1.39) *p* = 0.179
Informal/Primary	18 (27.3%)	48 (72.7%)	66	
Secondary/Higher	9 (42.9%)	12 (57.1%)	21	
Total	27 (31.0%)	60 (69.0%)	87	
3. Maternal Occupation.				1.00 (0.38–2.62) *p* = 1.000
Not Working	9 (31.0%)	20 (69.0%)	29	
Working	18 (31.0%)	40 (69.0%)	58	
Total	27 (31.0%)	60 (69.0%)	87	
4.Maternal Age (Years)				1.08 (0.43–2.67) *p* = 0.873
≤ 28	14 (31.8%)	30 (68.2%)	44	
≥ 29	13 (30.2%)	30 (69.8%)	43	
Total	27 (31.0%)	60 (69.0%)	87	
5.Maternal Religion.				0.98 (0.38–2.52) *p* = 0.974
Muslim	17 (30.9%)	38 (69.1%)	55	
Christian	10 (31.3%)	22 (68.8%)	32	
Total	27 (31.0%)	60 (69.0%)	87	
6.Maternal Marital Status.				0.40 (0.11–1.52) *p* = 0.274
Married/Cohabiting	22 (28.6%)	55 (71.4%)	77	
Single/Divorced	5 (50.0%)	5 (50.5%)	10	
Total	27 (31.0%)	60 (69.0%)	87	

Among the vitamin D deficient children, 19.8% were cases. Majority had informal or primary education, were of 2nd tertile socio-economic status, Muslims and either married or cohabiting. None of the factors studied showed any statistically significant difference between cases and controls in the vitamin D deficient children (Table 4).

Among vitamin D sufficient children, 31.0% were cases. Majority had informal or primary education, were of 2nd tertile socio-economic status, Muslims and either married or cohabiting. None of the factors studied showed any statistically significant difference between cases and controls in the vitamin D sufficient children (Table 5).

Binary logistic regression analysis was done to control for confounders between the different variables and diarrhoea in the children. Factors with a *p* value ≤ 0.2 in the univariate analysis were then entered into the regression model (Table 6).

**Table 6:** Binary Logistic Regression of Variables against Cases

Variables	Cases* (N – 185)	OR	95% CI	*P* Value
1 Vitamin D Status: Deficient	99	0.45	0.21–0.93	0.032
Reference: Sufficient	86			
2 Child Sex: Male	107	1.94	0.92–4.09	0.082
Reference: Female	87			
3 Child Nutritional Status: Severe Acute Malnutrition	66	2.61	1.26–5.39	0.010
Reference: Other Nutritional Status Combined	119			
4 Maternal Marital Status: Single/Divorced	17	1.23	0.37–4.07	0.735
Reference: Married/Cohabiting	168			
5 Maternal Education Level: Informal/Primary	126	0.68	0.28–1.64	0.391
Reference: Secondary/Higher	59			
6 Maternal Occupation: Not Working	74	0.80	0.38–1.70	0.562
Reference: Working	111			
7 Paternal Education Level: Informal/Primary	108	0.85	0.38–1.88	0.683
Reference: Secondary/Higher	77			

* 3 cases were not included in the analysis due to missing data for paternal education level

Binary regression analysis showed that a child with diarrhoea was 2.61 times more likely to have SAM, independent of other factors (95% CI: 1.06–5.28, p 0.036). Children with VDD were found to be less likely to have diarrhoea as compared to children without VDD. Other factors which were significantly associated with diarrhoea in univariate did not reach significance in the regression model i.e. sex of the child, maternal education, maternal occupation, maternal marital status and paternal education.

## Discussion

This study found a high prevalence of vitamin D deficiency in the study population with a majority 101 (53.7%) being vitamin D deficient, 64 (34.0%) insufficient and only 23 (12.2%) had sufficient levels of vitamin D. This finding is similar with the findings in other African countries such as; Cape Town, South Africa 62.7%, Guinea-Bissau, West Africa 39%; as well as other parts of the world which receive perennial sunshine like Costa Rica 28%, Tasmania, South Australia 68%, and Kuala Lumpur, Malaysia 35.3% [7, 15–18]. The High prevalence of VDD in Cape Town and Tasmania is most likely due to the fact that these areas are located outside the Tropics of Capricorn geographically, whilst the remaining listed geographical areas are located between the Tropic of Cancer and the Equator and therefore having longer exposure to sunshine and thus lower prevalence of VDD.

Overall comparison of the median vitamin D levels between cases and all controls showed that being VDD was associated with less incidence of diarrhoea. This is in sharp contrast to studies carried out in Columbia, Egypt, Pakistan and Saudi Arabia [9–12] which showed VDD to be associated with an increased incidence of diarrhoea. However, in the subgroup analysis the difference became apparent whereby the sick controls had a significantly lower median vitamin D levels than healthy controls and cases, which may explain this association when comparing cases to all controls in this study. This may imply that VDD is related to childhood illnesses and not specifically to diarrhoea or that the depletion of vitamin D becomes obvious post illness.

Logistic regression analysis which included socio-demographic factors possibly associated with vitamin D deficiency as well as diarrhoeal morbidity showed no significant association in children under five years of age. The finding did not correlate with studies carried out in Columbia, Egypt, Pakistan and Saudi Arabia [9–12] which showed VDD to be associated with an increased incidence of diarrhoea. However, nutritional status was found to be an important confounder between cases and the vitamin D outcome and this may be the cause of the lack of association with other factors in this study.

Furthermore, the logistic regression analysis showed that children with diarrhoea were 2.61 times more likely to have SAM as compared to those with without diarrhoea, and that having SAM was independently associated with having diarrhoea. This in keeping with the findings of the study carried out in Gambia, West Africa [19]. The other nutritional statuses (i.e. mild and moderate malnutrition) were not found to be associated with diarrhoea. The above is an expected finding for it is a well-known age-old association that SAM leads to diarrhoea, and that diarrhoea also leads to SAM [20].

A separate analysis among patients with vitamin D deficiency and those with vitamin D sufficiency showed that neither socio-economic status of the caregivers nor other maternal factors such as age, religious denomination, marital, educational or occupational statuses had any statistically significant difference between the cases and the controls of the respective groups. Concerning maternal education, the findings were in contrast to study in Indonesian children which showed there was a statistically significant association between maternal education and prevalence of diarrhoea [21].

Within the control groups, a sub-analysis showed that sick controls were 5.03 times more likely to be VDD than healthy controls, this was found to be statistically significant, previous studies have shown that low levels of vitamin D is associated with susceptibility and severity to acute and chronic illnesses [4, 5, 7, 8]. A plausible biological explanation would be that VDD is related to morbidity, and since it is more an immune-modulator rather than a gut epithelial function vitamin like vitamin A, in this study we see that sick children were significantly VDD.

This is a case-control study in a population which is relatively homogenous meaning that most associations are therefore likely to be significant. Furthermore, this is among the first few studies on vitamin D and diarrhoea done regionally and it gives a snap shot of the burden of VDD in the local setting. This case control study however was carried out in a single tertiary level hospital thus the results cannot be generalized to the community and other health facilities.

This study used cut-off values for vitamin D from the US Endocrine Society, where VDD has been defined as 25(OH) D less than 30 ng/ml. This cut-off has also been suggested by the National Osteoporosis Foundation and International Osteoporosis Foundation. This cut-off value has been used in most studies studying vitamin D status in under-five children. However, the Institute of Medicine has suggested different cut-off values, defining VDD as 25(OH) D below 20 ng/ml. The prevalence of VDD would have been lower in this study if more rigorous cut-off values would have been used.

## Conclusion

In conclusion, this study shows a high prevalence of vitamin D deficiency in the study population. This was not significantly different between those with diarrhoea and those without diarrhoea, however, those with other morbidities (sick controls) had 3.2 and 5 times higher risk of VDD compared to cases and healthy controls respectively. Moreover, severe acute Malnutrition was independently associated with having diarrhoea.

Further studies are required to investigate the cause of the high prevalence of VDD in children and in particularly sick children. In addition, large cohort studies in the community are needed to determine possible temporal relationships of Vitamin D deficiency and morbidity including diarrhoea.

## Data Availability

The datasets used and/or analysed during the current study are available from the corresponding author on reasonable request.

## Abbreviations

DD: Diarrhoeal Diseases
IMCI: Integrated Management of Childhood Illness
MNH: Muhimbili National Hospital
MUHAS: Muhimbili University of Health and Allied Sciences
SAM: Severe Acute Malnutrition
SES: Socio-economic Status
SPSS: Statistical Package for the Social Sciences
THDS: Tanzania Health and Demographic Survey
UV B: Ultraviolet B radiation
VDD: Vitamin D deficiency
Vit. D2: Ergocalciferol
Vit. D3: Cholecalciferol
WHO: World Health Organization
